# Exploring the acquisition and production of grammatical constructions through human-robot interaction with echo state networks

**DOI:** 10.3389/fnbot.2014.00016

**Published:** 2014-05-06

**Authors:** Xavier Hinaut, Maxime Petit, Gregoire Pointeau, Peter Ford Dominey

**Affiliations:** ^1^Stem Cell and Brain Research Institute, INSERM U846Bron, France; ^2^Université de Lyon, Université Lyon ILyon, France; ^3^Centre National de la Recherche ScientifiqueBron, France

**Keywords:** language acquisition, human-robot interaction, language production, grammatical constructions, recurrent neural networks, iCub humanoid, reservoir computing, anytime processing

## Abstract

One of the principal functions of human language is to allow people to coordinate joint action. This includes the description of events, requests for action, and their organization in time. A crucial component of language acquisition is learning the grammatical structures that allow the expression of such complex meaning related to physical events. The current research investigates the learning of grammatical constructions and their temporal organization in the context of human-robot physical interaction with the embodied sensorimotor humanoid platform, the iCub. We demonstrate three noteworthy phenomena. First, a recurrent network model is used in conjunction with this robotic platform to learn the mappings between grammatical forms and predicate-argument representations of meanings related to events, and the robot's execution of these events in time. Second, this learning mechanism functions in the inverse sense, i.e., in a language production mode, where rather than executing commanded actions, the robot will describe the results of human generated actions. Finally, we collect data from naïve subjects who interact with the robot via spoken language, and demonstrate significant learning and generalization results. This allows us to conclude that such a neural language learning system not only helps to characterize and understand some aspects of human language acquisition, but also that it can be useful in adaptive human-robot interaction.

## Introduction

### Issues in language acquisition

The ability to learn any human language is a marvelous demonstration of adaptation. The question remains, what are the underlying mechanisms, and how do humans make the link between the form of a sentence and its meaning? Enormous debate has ensued over this question. The debate can be characterized with one end of the continuum, Piaget's constructivism, holding that language can be learned with general associative mechanisms, and the other end, Chomsky's innatism, holding that the stimulus is so poor, that language could only be learned via a highly specialized universal grammar system (Piattelli-Palmarini, 1980). We and others have argued that linguistic environment is rich—in response to the “Poverty of stimulus hypothesis” (reviewed in Dominey and Dodane, 2004). As the child is situated in the environment, it has access to massive non-linguistic information that can aid in constraining the possible meanings of phonemes, words or sentences that it hears (Dominey and Dodane, 2004). In this context, social interaction is clearly an important factor that helps the child to acquire language, by focusing its attention on the same object or event as the person he is interacting with via joint attention. Joint attention permits one to considerably reduce the possible mappings between what is said and what is happening in the environment. Joint attention happens sufficiently often to assume it as one of the reliable ways to help the child to acquire language: for instance when playing a game, showing an object, ritualized situations including bathing and feeding, etc. (Carpenter et al., 1998; Ricciardelli et al., 2002; Tomasello, 2003; Dominey and Dodane, 2004; Sebanz et al., 2006; Knoblich and Sebanz, 2008; Tomasello and Hamann, 2012).

Despite the potential aid of joint attention, mapping the surface form onto the meaning (or deep structure) of a sentence is not an easy task. In a first step in this direction, Siskind demonstrated that simply mapping all input words to all possible referents allows a first level of word meaning to emerge via cross-situational statistics (Siskind, 1996). However, simply associating words to specific actions or objects is not sufficient to take into account the argument structure of sentences in language. For instance given these two sentences “Mary hit John.” and “John was hit by Mary.” which have the same meaning but with a different focus or point of view, how could a purely word-based system extract the exact meaning of the sentence? How could an infant determine who is doing the action (the *agent*) and who endures the action (the *object*)? As simple this example is, relying only on the semantic words, and their order in the sentence, will not permit to reliably distinguish the *agent* from the *object*.

To begin to answer this question, we consider the notion of grammatical construction as the mapping between a sentence's form and its meaning (Goldberg, 1995, 2003). Goldberg defines constructions as “stored pairings of form and function, including morphemes, words, idioms, partially lexically filled and fully general linguistic patterns” (Goldberg, 2003). Constructions are an intermediate level of meaning between the smaller constituents of a sentence (grammatical markers or words) and the full sentence itself.

**Figure 1:**
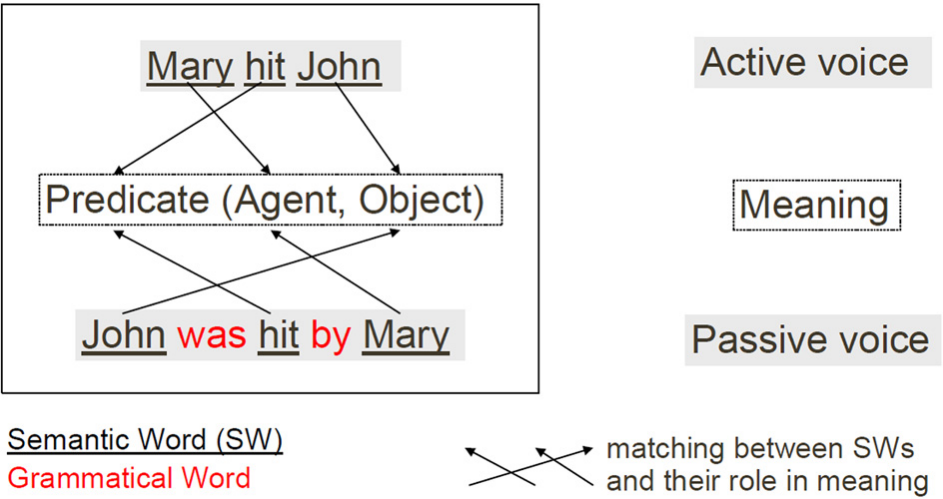
**Schematic characterization of the thematic role assignment task**. Solving this task consists in finding the adequate mapping between the content words (i.e., semantic words) and their roles in the meaning of a given sentence. This mapping is represented by the set of arrows (here three) for each sentence surface-meaning mapping.

Typical grammatical constructions could be used to achieve thematic role assignment, that is answering the question “Who did what to whom.” This corresponds to filling in the different slots, the roles, of a basic event structure that could be expressed in a predicate form like *predicate(agent, direct object, indirect object*, or *recipient)*. A simplified summary of characterization of grammatical constructions can be seen in Figure 1.

Solving the thematic role assignment problem consists in finding the correct role for each semantic word (i.e., content word or open class word). It thus consists in finding the *predicate*, the *agent*, the *object*, and the *recipient* for a given action. In the preceding example this means that *hit* is the predicate, *Mary* is the agent and *John* is the object. How could one use grammatical constructions to solve this thematic role task for different surface forms as illustrated in Figure 1? According to the cue competition hypothesis of Bates and MacWhinney (Bates et al., 1982; Bates and MacWhinney, 1987) the identification of distinct grammatical structures is based on combinations of cues including grammatical words (i.e., function words, or closed class words), grammatical morphemes, word order and prosody. Thus, the mapping between a given sentence and its meaning could rely on the ordered pattern of words, and particularly on the pattern of function words and markers (Dominey, 2003; Dominey et al., 2003). As we will see in the Materials and Methods section, this is the assumption we make in the model in order to resolve the thematic role assignment task, that is, binding the sentence surface to its meaning. In English, function words include “the,” “by,” “to”; grammatical markers include verb inflexions “-ing,” “-ed,” or “-s.” One interesting aspect of grammatical words and markers is that there are relatively few of them, compared to the potentially infinite number of content words (i.e., semantic words). Hence the terms “closed class” for grammatical words and “open class” for semantic words. As these closed class words are not numerous and are often used in language, it could be hypothesized that children would learn to recognize them very quickly only based on statistical speech processing. This argument is reinforced by the fact that such words or markers are generally shorter (in number of phonemes) than content words. This notion of prosodic bootstrapping (Morgan and Demuth, 1996) is reviewed and modeled in Blanc et al. (2003).

### Overview of the tasks

In this study we investigate how a humanoid robot can learn grammatical constructions by interacting with humans, with only a small prior knowledge of the language. This includes having a basic joint attention mechanism that allows the robot to know for instance what is the object of focus. We approach our simplified study of language acquisition via two conditions: language comprehension and language production. Both conditions will have two modes: a training mode, when the human acts as a kind of teacher, and a testing mode, where the human could test the language capabilities of the robot as in child-caregiver interactions. The experimental tasks will test the ability of our neural network model of language acquisition to understand and to produce meaningful language.

We have shown in previous studies that the neural model used (1) can learn grammatical constructions correctly generated with a context-free grammar (with one main and one relative clause), (2) can show interesting performance in generalizing to not learned constructions, (3) can show predictive activity during the parsing of a sentence and in some cases give the final correct parse before the sentence ended, and (4) that the neural activity may be related to neurophysiological human recording (Hinaut and Dominey, 2012, 2013). We believe that these results demonstrate that the model may be suitable to a developmental robotic approach, extending our previous work in this domain (Dominey and Boucher, 2005a,b).

Here we have four goals: (1) to determine if it is possible to use the model in an interactive fashion with humans, that is, to integrate this neural model in the robotic architecture and make it communicate and work in real-time with the other components of the architecture (speech recognition tool, etc.); (2) test the model in a productive manner, that is instead of “understanding” a sentence, it will be able to produce one, that is, to produce the sequence of words of the grammatical structure given the thematic roles and the sentence type (canonical or non-canonical); this has not been done in our previous experiments with the neural model; (3) in the comprehension task, test if the neural model can learn constructions that allow for commands that manipulate the temporal structure of multiple events. For instance to correctly respond to the sentence “before you put the guitar on the left put the trumpet on the right.” Finally, (4) we test the model with language input from naïve subjects, in order to determine if indeed this adaptive approach is potentially feasible in less structured environments.

In the Material and Methods section we will first briefly present the robotic platform and the interaction environment. We will then describe the two neural models used for the comprehension and production tasks. Finally, the integration of these components will be presented. In the Experiment section we will describe the experimental procedures for the *scene describer* task, and the *action performer* task. In Results section we will illustrate the functioning of the system in these two modalities, including figures illustrating the human-robot interactions, and figures illustrating typical neural activation recorded for both models. We then present the data and learning and generalization results for an extended experiment with five naïve subjects. In the last section, we will discuss the results and interesting aspects that the combination of a comprehension and production neural models provide. Training and testing data used in the experiments, and corresponding to the figures showing the output neural activity of the models are provided in Appendices section.

## Materials and methods

### iCub platform and interaction architecture

**Figure 2:**
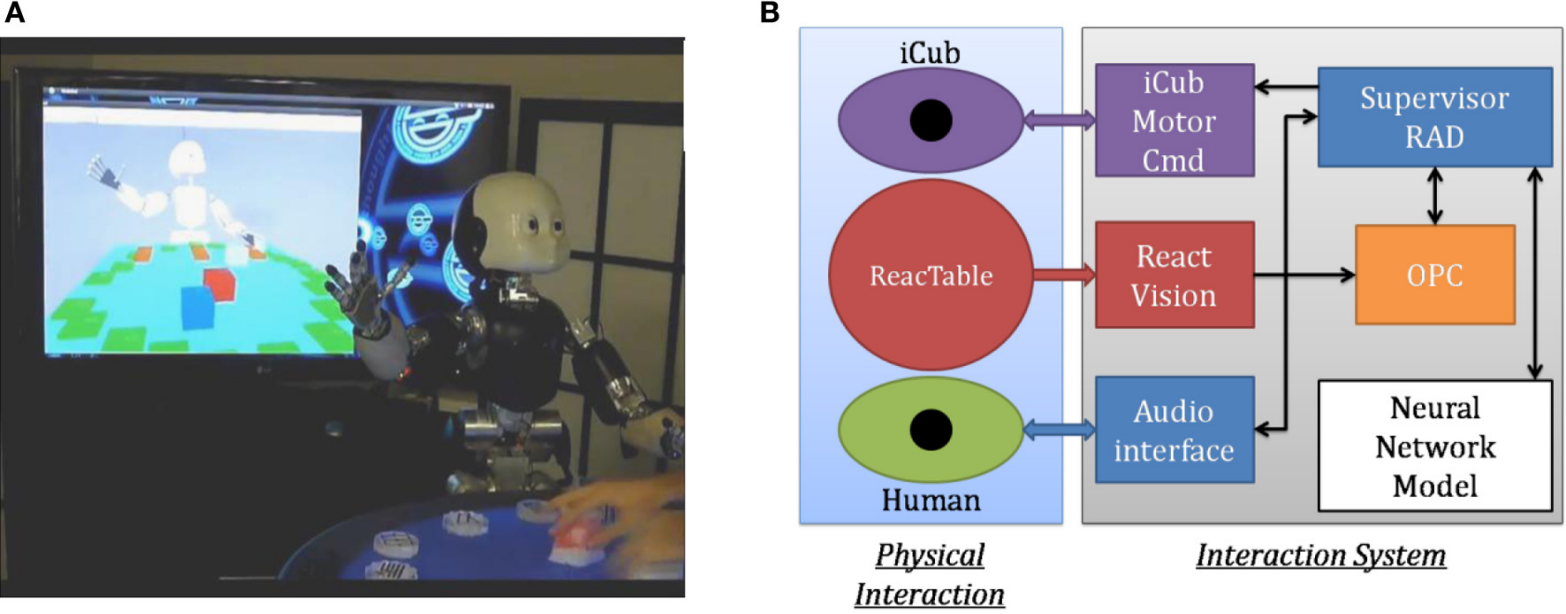
**Robotic Platform. (A)** iCub humanoid robot with the ReacTable. **(B)** System architecture overview. The Supervisor coordinates all interactions between the human and the different components of the system. When the human moves an object on the ReacTable, the coordinates are transformed into the robot space, and stored in the Object Properties Collector (OPC). For Action Performance when the human speaks, the words are recognized by the audio interface, then they are packaged and sent to the Neural Network by the Supervisor. Resulting commands from the Neural Network are processed and forwarded to the iCub Motor Command (iCub Motor Cmd) interface by the Supervisor, the robot then performs the given actions. For Scene Description, the Cartesian coordinates of the objects are transmitted from the OPC to the Supervisor. Spatial relations between “environmental” objects and the object of focus are computed. They are then sent to the Neural Network together with the sentence type (canonical or non-canonical). The sentence generated by the Neural Network is sent to the Audio interface for speech synthesis, again under the control of the Supervisor.

The platform that we used is the iCub, furnished by the FP6 EU consortium RobotCub (see Figure 2). The iCub (Metta et al., 2010) is a 53 DOF humanoid robot built by the Italian Institute of Technology (IIT) with the size of a three and a half year-old child. We use YARP (Metta et al., 2006) as the robotic middleware with the Biomimetic Architecture for Situated Social Intelligence Systems (BASSIS architecture) built for the FP7 Experimental and Functional Android Assistant project (Petit et al., 2013).

The Supervisor module is implemented with the CSLU RAD Toolkit (Sutton et al., 1998) Rapid Application Development for spoken language interaction. It uses the Festival system for speech synthesis (Taylor et al., 1998) and Sphinx II for spoken language recognition (Huang et al., 1993). The Supervisor provides a dialog management capability built as a finite-state system. This capability allows the user to guide the robot into the different states of behavior, but is distinct from the neural language model, described below. The Supervisor/Manager orchestrates the communication and exchange of data between speech recognition and synthesis, the neural models for language comprehension and generation, and the robot perception and action systems.

The ability of the iCub to perceive physical objects and their manipulation in the context of action performance and description is provided by the ReacTable, which detects objects on a translucid table based on detection of fiducial markers on the object bases, using an infra-red camera (Bencina et al., 2005). The ReacTable thus provides data on the type and position of objects on the table with high precision. The ReacTable is calibrated into the motor space of the iCub, so that object locations can be used for physical interaction.

The motor control for iCub reaching, grasping, and object manipulation is provided by DForC—Dynamic Force Field Controller—(Gori et al., 2012), based upon dynamic force control. The robot has a small set of primitive actions: put(object, location), grasp(object), point(object).

### Neural language model

The neural language processing model represents the continued development of our work based on the underlying concept of a recurrent network with modifiable readout connections for grammatical construction processing (Dominey, 2003; Dominey et al., 2003; Hinaut and Dominey, 2012, 2013). As described in the context of grammatical constructions above, for sentence processing we have shown that the pattern of open and closed class word order could be used to correctly identify distinct grammatical constructions and extract their meaning for a small set of sentences. More recently we have demonstrated the extension of this ability to larger corpora from several hundreds of uniquely defined construction-meaning pairs, to tens of thousands distinct constructions including redundant and ambiguous meanings (Hinaut and Dominey, 2013). As the neural model has anytime learning property, it is of interest to use it for exploring language acquisition in a developmental robotics perspective.

The core of the language model is a recurrent neural network, with fixed random connections, which encodes the spatio-temporal context of input sequences. This sequence-dependent activity then projects via modifiable connections to the read-out layer. Modification of these read-connections by learning allows the system to learn arbitrary functions based on the sequential input. This framework has been characterized as Reservoir Computing (Verstraeten et al., 2007; Lukosevicius and Jaeger, 2009), where the recurrent network corresponds to the reservoir, and has been developed in different contexts. The first expression of the reservoir property with fixed recurrent connections and modifiable readout connections, was developed in the context of primate neurophysiology, with the prefrontal cortex as the reservoir, and modifiable cortico-striatal connections as the modifiable readout (Dominey, 1995; Dominey et al., 1995). Further development was realized in related systems including the Liquid State Machine (Maass et al., 2002), and Echo State Network (Jaeger, 2001; Jaeger and Haas, 2004).

The model employed in the current research (Hinaut and Dominey, 2013) pursues this parallel between brain anatomy and the reservoir computing framework. Prefrontal cortex is modeled as a recurrent network that generates dynamic representations of the input, and striatum as a separate population connected to cortex via modifiable synapses, which learns to link this dynamic representation with a pertinent output. Cortex and striatum corresponding respectively to the reservoir and readout. The reservoir is composed of leaky neurons with sigmoid activation. The following equation describes the internal update of activity in the reservoir:

(1) $$x(t+1)=(1-\alpha )x(t)+\alpha f\left(W_{res}x(t)+W_{in}u(t+1)\right)$$

where *x*(*t*) represents the reservoir state; *u*(*t*) denotes the input at time *t*; α is the leak rate; and *f*(·) is the hyperbolic tangent (tanh) activation function. W*in* is the connection weight matrix from inputs to the reservoir and W*res* represents the recurrent connections between internal units of the reservoir. In the initial state, the activation of all internal units of the reservoir is zero. The inverse of the leak rate (1/α) could be interpreted as the time constant of the system.

By definition, the matrices W*in* and W*res* are fixed and randomly generated. Internal weights (W*res*) are drawn from a normal distribution with mean 0 and standard deviation 1 and then rescaled to the specified spectral radius (the largest absolute eigenvalue of the W*res* matrix). The input weight matrix W*in* was first generated with values chosen randomly between −1 and 1 with a 50% probability. The W*in* matrix was then rescaled depending on the experiment (*input scaling* parameter). The density of the input connections is 100%.

The output vector of the system which models the striatum is called the readout. Its activity is expressed by the following equation:

(2) $$y(t)=W_{out}x(t)$$

with W*out* the matrix of weights from the reservoir to the readout (output). The activation function of readout units is linear. Interestingly, the readout activity gives a pseudo-probabilistic response for each output unit. To train the read-out layer (i.e., compute W*out*), we use a linear regression with bias and pseudo-inverse method (Jaeger, 2001). This general model is applied in two distinct instantiations. One model processes commands (sentences) and generates a predicate-argument representation of the meaning. The second describes observed actions, i.e., given a predicate-argument meaning as input, it generates a sentence describing that meaning. Thus, the comprehension system learns to map semantic words of input sentences onto an output that characterizes the role (action, agent, object, recipient) of each of these semantic words, based on the structure of grammatical words in the sentence. The production system learns the inverse mapping, from the meaning (i.e., specification of the role of each semantic word) onto a sentence form.

#### Comprehension model for action performing task

**Figure 3:**
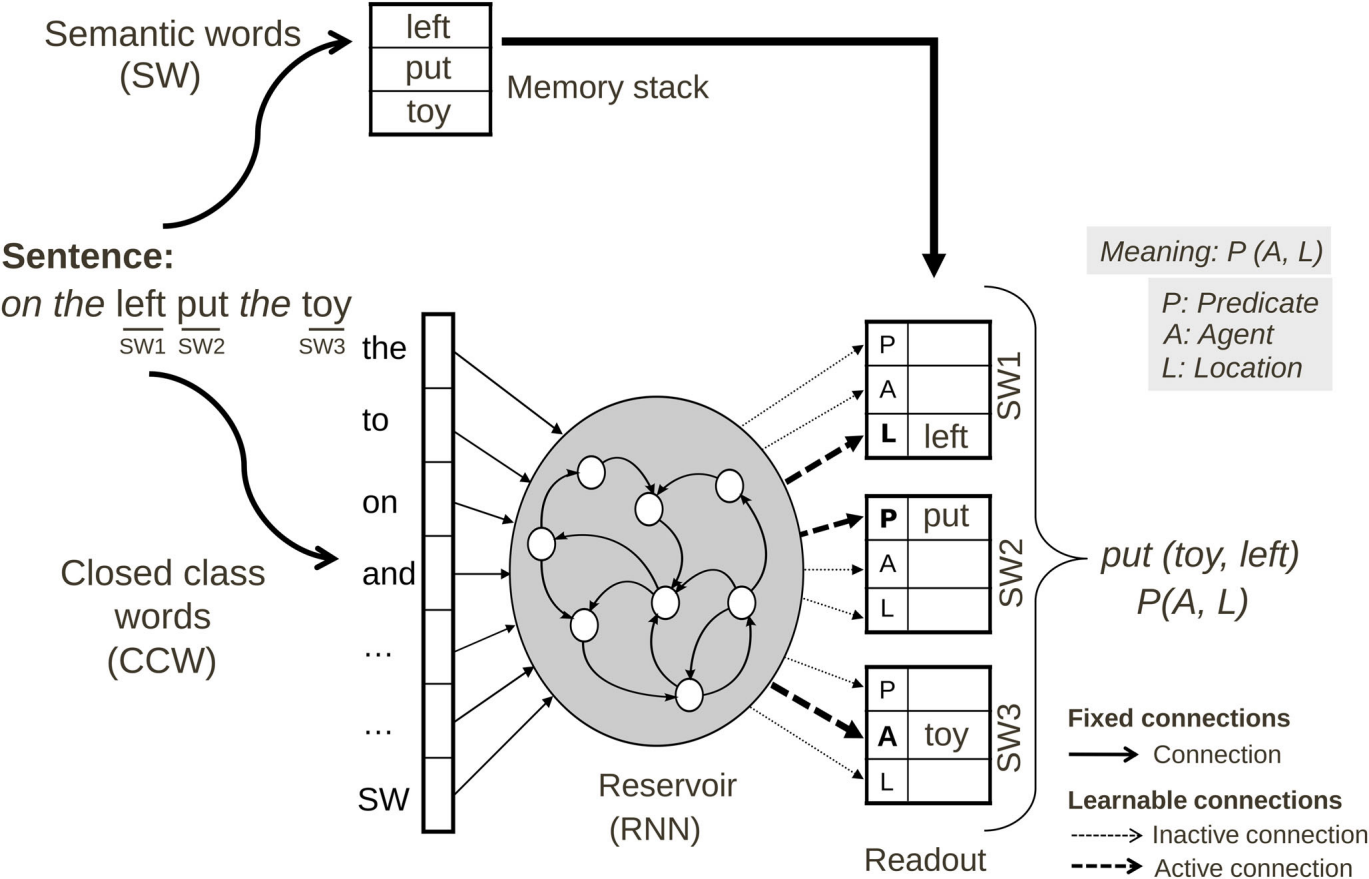
**Neural comprehension model for the Action Performing task**. Sentences spoken by the user are first transformed into grammatical forms, i.e., all semantic words (SW) are replaced by a SW marker. The reservoir is given the grammatical form word by word. Each word activates a different input unit. Based on training, the readout connections from the reservoir provide the coding of the predicate-argument meaning in the readout neurons, thus forming the grammatical construction as a mapping from grammatical form to meaning. The meaning of an input sentence is specified in terms of the role (predicate, agent or location) for each semantic word SW.

The architecture of the comprehension model is illustrated in Figure 3.

***Preprocessing.*** Before being provided as input to the neural model, the sentence must first be transformed by extracting the open-class (i.e., semantic) words. The resulting grammatical form is characterized by the sequential pattern of closed-class (i.e., grammatical) words. This operation is performed by replacing all open class words by “SW” markers (SW: semantic word). The semantic words removed from the sentence are stored in a working memory. The working memory acts as a first-in-first-out (FIFO) stack: the words will be retrieved in the same order as in the output. For example, when semantic word 2 (SW2) is determined by the model to be the agent, the actual word corresponding to SW2 will be retrieved as the agent of the described action. The closed class words used were: “after,” “and,” “before,” “it,” “on,” “the,” “then,” “you.”

***Reservoir parameters.*** The number of unit used in the reservoir is 100. The leak rate used is 1/6 (= 0.1666…). The *input scaling* is 0.75. The *spectral radius* is set to 1.

***Sentence input parameters.*** Given an input sentence, the model should assign appropriate thematic roles to each semantic word. The presentation of inputs is illustrated in Figure 3. Sentences are represented in the input as grammatical forms, where specific instances of noun and verb words (semantic words—SW) are replaced by a “SW” marker. Thus, a given grammatical construction can code for multiple sentences, simply by filling in the “SW” markers with specific words. In this way of coding, the reservoir cannot distinguish between nouns or verbs, as they have the same input neuron. This is an interesting characteristic when using the model within a robotic platform, because when sentences are processed there is no need to do a preprocessing in order to classify words as nouns or verbs.

The total number of input dimension is 9; 8 for closed class words, 1 for the semantic word marker. Each word is coded as a square wave of 1 time step. There is no pause between successive word presentations (the use of pauses does not have significant influence on the results), but there is a final pause at the end of the sentence in order to inform the model that the sentence is finished. This final pause could be replaced by a period, as it would have the same function as a terminal symbol. An offset of the sentence was added at the beginning of the inputs if they were not of maximal length, in this way the correct final meaning is always given at the last time step.

***Desired meaning output coding.*** Making the analogy with an infant who is learning language in the presence of sentences and their corresponding meanings, we consider that the system is exposed to a meaningful scene while the input sentence is being presented. Thus, the system has access to the meaning starting at the beginning of the presentation of the sentence, hence the desired output teacher signal is provided from the beginning of the first word until the end of the input. All the output neurons coding the meaning are clamped at 1, all other output neurons are clamped to 0. By forcing the correct outputs to be 1 from the onset of the sentence during learning, we obtain predictive activation when processing (i.e., testing) a sentence after the learning phase. This can be seen in the results section in Figure 8, below (see Hinaut and Dominey, 2011, 2013, for more details). The meaning output dimension is 36 (=6 × 3 × 2): 6 semantic words that each could have three possible thematic role assignment (predicate, agent or location), for each of up to maximum 2 verbs.

***Post-processing.*** To determine the meaning specified in the output, the activity of the output at the last time step is thresholded. For each SW, we take the role that has the maximum activation (if there are several). Each semantic word in the FIFO stack is then bound with its corresponding role(s). The full predicative meaning is then obtained and written in the output data file in order to be processed by the Supervisor module, and then used to command the robot.

#### Production model for scene description task

**Figure 4:**
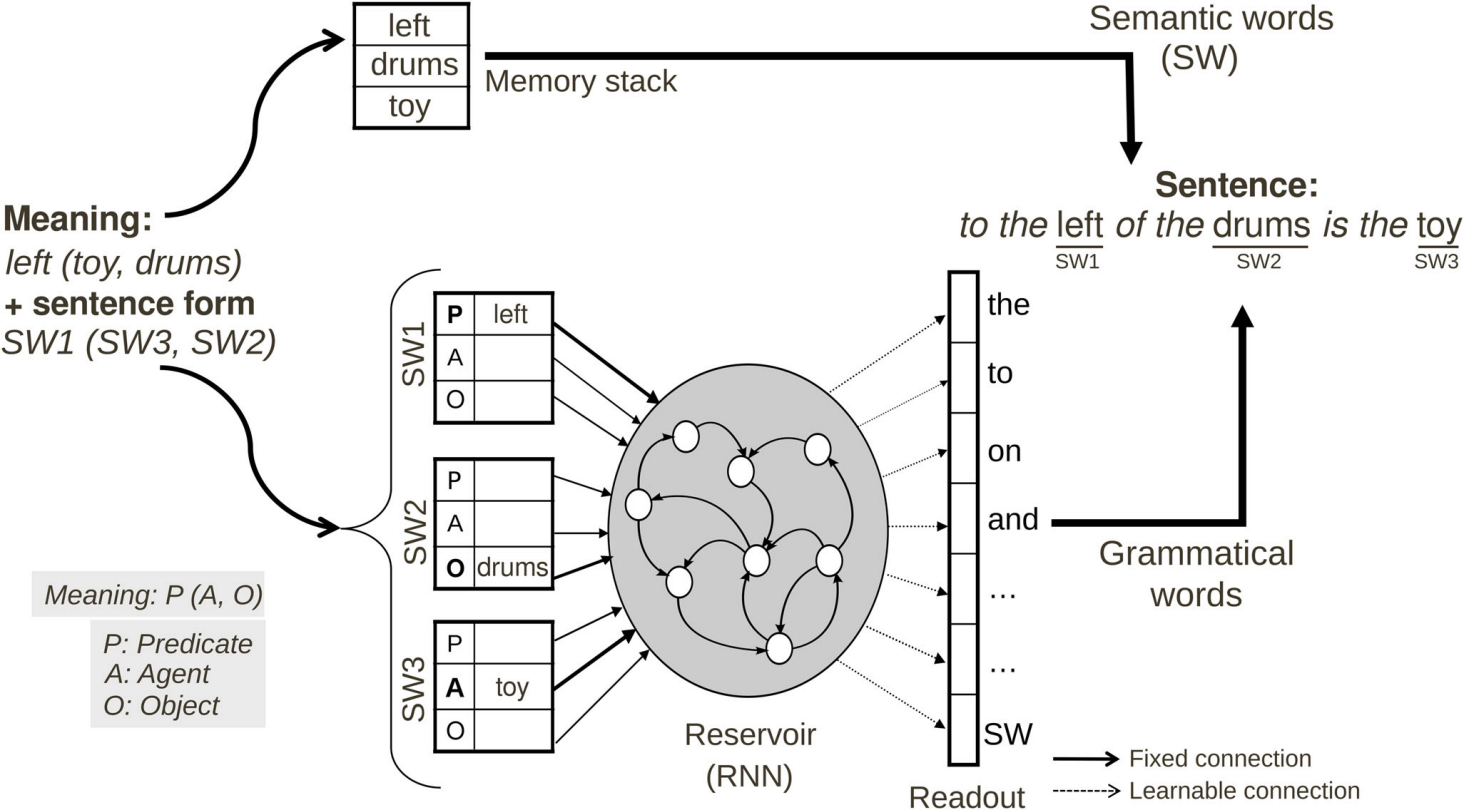
**Neural production model for Scene Description task**. The input has 2 components: (1) meaning format <Predicate(Agent, Object)—left(toy, drums)> corresponding to relation *toy to the left of drums*, and (2) construction format with <SW1—Predicate, SW2—Object, SW3—Agent> which could be written in a compact way as SW1(SW3, SW2). The full input information could be represented as <SW1_Predicate—left>, <SW2_Object—drums>, and <SW3_Agent—toy>. The system must find a construction that allows this mapping of SWs to thematic roles. SW#_θ: Semantic Word # has thematic role θ, with # the relative position in the sentence among all Semantic Words.

We have described the functioning of the language model that learns to map input sentences onto a predicate-argument representation of their meaning. Now we consider the reverse case, where given a meaning, the model should produce a sentence. This model thus employs the same principals as the language comprehension model, but we now perform the reverse operation—from a meaning we want to generate the corresponding sentence (see Figure 4). It is important to recall that there are potentially multiple possible sentences for describing a given scene or meaning (as illustrated in Figure 1). To resolve this ambiguity, we provide additional input to the model, to indicate if we want a canonical (e.g., standard, active voice) or a non-canonical (e.g., passive voice).

***Preprocessing.*** The model is given the meaning and the sentence type desired (canonical or non-canonical) by the Supervisor module. This information is converted in the corresponding coded meaning, as described in Figure 4. The semantic words of the meaning are stored in the FIFO memory.

***Reservoir parameters.*** The number of units used in the reservoir is 500. The leak rate used is 0.75. The *input scaling* is set to 0.01. The *spectral radius* is set to 2.

***Input and output coding.*** The coded meaning is given, for all the input units concerned, as a constant input activation set to 1. Remaining input units are set to 0. This is consistent with the output representation of the meaning in the first model presented in Comprehension Model for Action Performing Task (comprehension model). As illustrated in Figure 4 the desired mapping of the open class words onto thematic roles is specified by activating the appropriate input neurons. The input activation lasts during all the input presentation. The input dimension is the same as the output dimension of the comprehension model 6 × 3 × 2 = 36: 6 semantic words that each could have three possible thematic role assignment (predicate, agent or object), and each could have a role with at maximum two verbs. Table 1 illustrates how different coded-meanings can be specified for the same input meanings. This allows us to specify in the input if the sentence should be of a canonical or non-canonical form.

**Table 1:** **Representation and form of canonical and non-canonical sentences**.

	**Meaning**	**Sentence**	**Coded-meaning**
Canonical	Left(toy, drum)	The toy is left of the drums	SW2(SW1, SW3)
Non-canonical	Left(toy, drum)	To the left of the drums is the toy	SW1(SW3, SW2)
Double canonical	Left(violin, trumpet); right(violin, trumpet)	The violin is to the left of the trumpet and to the right of the guitar	SW2(SW1,SW3); SW4(SW1, SW5)
Double non-canonical	Left(violin, trumpet); right (violin, guitar)	To the left of the trumpet and to the right of the guitar is the violin	SW1(SW5,SW2); SW3(SW5, SW4)

Both examples of each single or double type have the same meaning. The sentences are different, and the mapping of semantic words onto the thematic roles in the meaning is different, as specified in the coded-meaning or sentence form. The semantic word that is the grammatical focus changes between canonical and non-canonical sentences. Both Meaning and Coded-meaning use the convention Predicate(Agent, Object). SW#: Semantic Word #, with # the relative position in the sentence among all Semantic Words.

Activation of the output units corresponds to the successive words in the retrieved construction. The closed class words used were: “and,” “is,” “of,” “the,” “to,” “.” (dot). The dot is optional and was not used for the experiments shown in Figure 9; it could be used in the future if several sentences have to be produced. The total number of output dimension is 7: 6 for closed class words and one for the SW marker.

The output teacher signal is as the following: each word is coded as a square wave of 5 time steps. Each word was separated with a pause of 5 time step. We used 5 time steps for each word and a pause of same duration between them in order to have an output activity that last a sufficiently long time; in this way each word could be discriminated more easily in the post-processing process. There is a final pause at the end of the teacher signal. All the teacher signals were of maximal length corresponding to the longest sentence.

***Post-processing.*** Once again, the output activity is first thresholded. Then each time an output exceeds the threshold, the corresponding word is added to the final construction (if the activity of this word last 4 or 5 time steps above the word it is considered only once). If several outputs are above the threshold, the word of maximal value is kept. Finally, the sentence is reconstructed replacing the SW makers with the semantic words kept in memory.

### Integrated system

**Figure 5:**
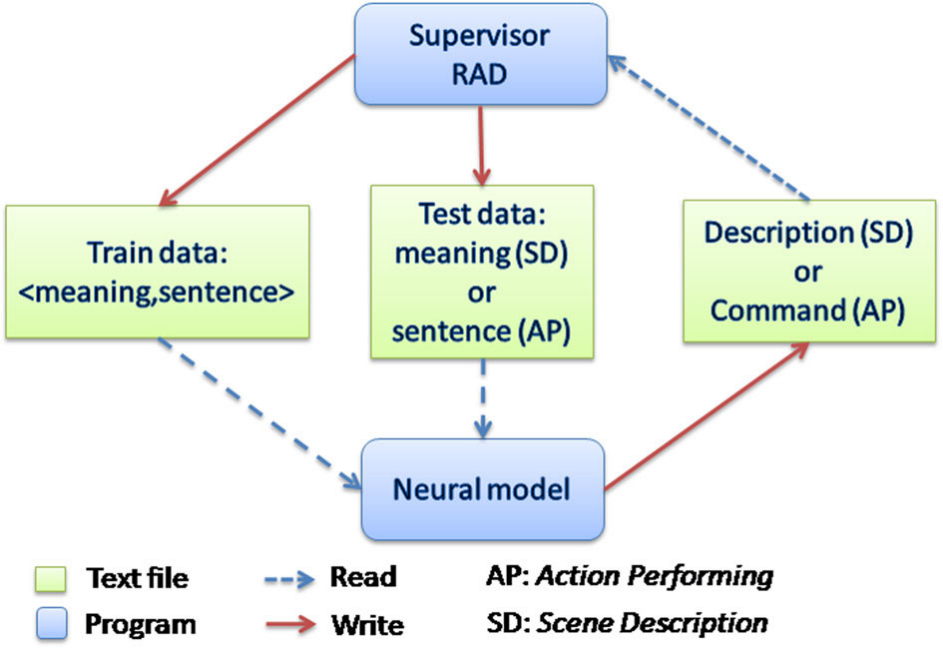
**Communication between modules**. The Supervisor manages the spoken interaction with the user and controls the robotic platform, providing different behaviors in SD and AP tasks. Depending on the mode selected by the user, train or test, it launches the neural model or not. In the train mode, pairs of <meaning, sentence> are stored in the train data file. In test mode, the sentence to be tested is written in the test data file, and both train and test files are sent at once to the Neural model. See Figure 2 for complementary information.

The system operates in real-time in a human-robot interaction. Figure 5 shows how the communication between modules is performed. Again, the system can operate in “action performer” (AP) and in “scene description” (SD) tasks, and the Supervisor module allows the user to specify which of these tasks will be used. The Supervisor interacts with the human through spoken language to determine if he wants to run the system in train mode—to teach the robot new <meaning, sentence> pairings—or in test mode—to use the corpus of pairings already learned by the robot. Thus, there are two tasks (AP or SD), each of which can be run in two execution modes (train or test). Details for AP and SD tasks are provided in the next section. Now we briefly describe train and test modes.

In train mode, the Supervisor incrementally generates one of the two training data files depending on the task (AP or SD). The human speech is transformed into text via the speech-to-text tool, and the meaning is given by the robotic platform (from perception or action). The <meaning, sentence> pairing is then written in the training data text file. In order to avoid populating the training files with bad examples in case of incorrect speech recognition, before writing the file the Supervisor asks the user for a verification (e.g., if it correctly understood the meaning). If the user wants the example to be added to the data file he answers “yes,” otherwise he answers “no.”

In test mode, the Supervisor processes the test example given by the user: in AP task the example is a sentence; in the SD task the example is a meaning (i.e., the user places objects in particular positions relative to the object of focus). This test example is a half-pairing of a complete sentence-meaning pair. First, the Supervisor generates a file containing the previously established training data, and the test example. It then launches the corresponding neural model (comprehension or production) depending on the task (respectively AP or SD). The neural model is trained with the training data, and then it processes the test half-pairing and generates the “missing half” in a text file. The Supervisor processes the file returned by the neural model and executes the action in the AP task or produces the sentence in the SD task.

## Experiments

We now illustrate in detail how the system works in two distinct modes: training and testing for the AP and SD tasks. An overview is provided in Table 1. In both tasks, meanings are expressed in a predicate-argument form: for instance *put(toy, left)* (for Action Performing task; see Figure 3), or *left(toy, drums)* (for the Scene Description task; see Figure 4). During training, meaning is produced by transforming the events and relative position of objects into the respective action and scene meanings. This is achieved by analyzing the change in object positions on the ReacTable (in order to get scene meanings) and by interrogating the program generating random robot action (for action meanings). Spoken sentences are transformed from a speech record into a list of words (using the Sphinx II recognizer) and paired with the associated meaning to populate the training database. The training mode is responsible for building a corpus of <sentence, meaning> pairs which will be fed to the neural model in order to train it. The human is then invited to build the database by interacting with the robotic platform. The type of interaction is different according to the task, AP or SD, as indicated in Table 2. In testing mode, the human provides one component of a <sentence, meaning> pair, and gets the missing component of the pair in return.

**Table 2:** **Summary of events in training and testing modes for the action Performer (AP) and scene describer (SD) tasks**.

	**Action performer (AP)**	**Scene describer (SD)**
Training	(1) Robot generates random action(s) [meaning]	(1) Human arranges objects on the table [meaning]
	(2) Human says a corresponding command [sentence]	(2) Human describes the scene [sentence]
Testing	(1) Human says a command [sentence]	(1) Human arranges objects on the table [meaning]
	(2) Robot performs corresponding action(s) [meaning]	(2) Robot describes the scene [sentence]

In brackets is indicated the half-pairing generated corresponding to each event.

### Experiment scenario 1: Action performing task

In the following X–Z are arbitrary objects (e.g., guitar, trumpet, violin), and, L and R are different locations (e.g., left, right, middle). In the training mode, one or two random action(s) are generated by the iCub using available objects (e.g., <put X on the R>, <grasp Y, point Z>, …). This produces the *meaning*. At the same time, the human user observes and then says the order (i.e., command) which, according to him, should command the robot to perform the(se) action(s): this corresponds to the *sentence*. The <*sentence, meaning*> pair can thus be constructed. The robot continues to randomly select possible actions and execute them, and the user provides the corresponding command, thus populating the database with <sentence, meaning> pairs.

In testing mode, the system uses the data generated in the learning mode in order to fully interact with the human, whereas in the training mode the system is more passive. In the Action Performing task the human says a command to the robot (providing the *sentence*). This test sample is passed to the neural model (Figure 3). The neural model produces the corresponding *meaning*, which is sent back to the Supervisor which translates the meaning into the corresponding robot command(s). The robot then produces the desired action(s).

### Experiment scenario 2: Scene description task

During the training phase for Scene Description task the user puts several objects on the table and specifies the focus object. Then he describes orally one or two spatial relations relative to the focus object (e.g., <the X is to the L of Y and to the R of Z>, …), providing the *sentence*. The Supervisor then uses the coordinates of the objects and the knowledge of the focus objects to find the relationships between the focus element and the other element(s) on the table, providing the *meaning*.

During the testing phase for the Scene Description task the user puts some objects on the table in a particular spatial relation, producing the *meaning*. This test example is passed to the neural model. The latter produces the corresponding *sentence* that is sent back to the Supervisor which produces the sentence via the audio interface (text-to-speech tool).

For both tasks during testing phase the data file that is transmitted to the neural model contains both the testing data and the training data. This permits to avoid executing the neural model each time one example is learned. Thus, the model learns the whole data set and then applies this to the test data (on which it is not trained).

### Experiment scenario 3: Naïve subject action performer task

In order to test the robustness of the system, we tested learning and generalization with data produced by five naïve subjects. In order to standardize the experiment we made a movie of a human performing a set of behaviors: 5 single actions and 33 double actions. For instance <*point(guitar)*> is an example of a single action: a corresponding sentence could be “Point to the guitar”; And <*point(guitar), put(toy, left)*> is an example of a double action: a corresponding sentence could be “Point to the guitar then put the toy on the left.” For each behavior (i.e., for each scene of the movie), we asked the subjects to give a “simple” command, and then a more “elaborate” one corresponding to the observed action(s), as if they wanted a robot to perform the same action. The subjects looked at the same scene twice, once before giving a “simple” command (i.e., order), and once before giving an “elaborate” one. Subjects saw each scene twice in order to obtain more spontaneous responses from them. Thus, subjects do not have to remember the scene and try to formulate both simple and elaborate sentences in a row. This resulted in a corpus of 5 (subjects) × 38 (behaviors) × 2 (canonical and non-canonical) = 380 sentences. The <sentence, meaning> corpus was obtained by joining the corresponding meanings to these sentences. Once this corpus was obtained, first, in order to assess the “learnability” of the whole corpus, we trained and tested the neural model using the same data set. Then generalization capability was tested using leaving-one-out method (i.e., cross validation with as many folds as data examples): for each <sentence, meaning> pair, the model was trained on the rest of the corpus, and then tested on the removed <sentence, meaning> pair.

## Results

### Human robot interaction

The iCub robot learns in real-time from human demonstration. This allows the robot to (1) perform complex actions requested by the user, and (2) describe complex scenes. Here “complex” means multiple actions with temporal (chronological) relations. The system can for instance execute commands like: “Before you put the guitar on the left put the trumpet on the right.” We demonstrate how this form of temporally structured grammatical construction can be learned and used in the context of human-robot cooperation.

**Figure 6:**
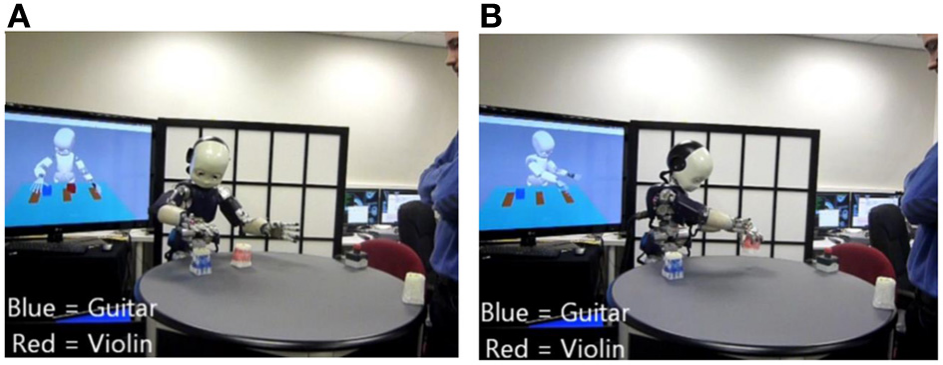
**Action Performing task**. The robot is performing the motor commands corresponding to the sentence “Point the guitar before you put on the left the violin.” **(A)** the robot is pointing “guitar” (blue object), **(B)** the robot is finishing the displacement of the “violin” (red object).

**Figure 7:**
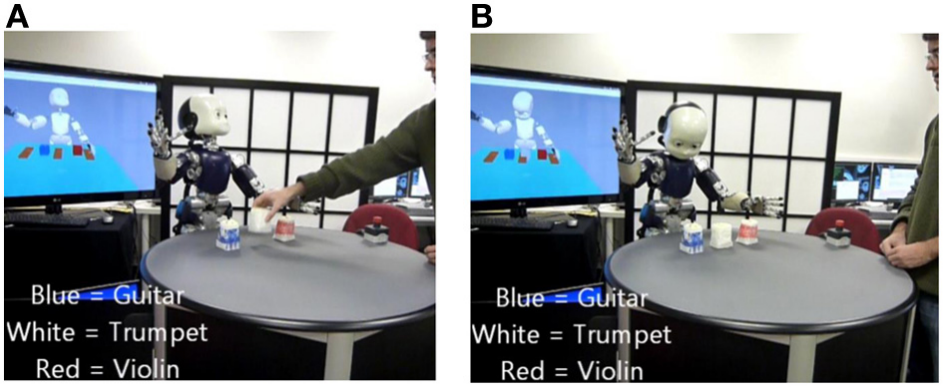
**“Scene Description” condition**. The robot have to describe the scene relative to the object of focus: **(A)** the user sets the object of focus in the scene, where other objects are already present; **(B)** the robot is describing the position of the focus object relative to the other objects.

In Figures 6, 7, we can see images extracted during human-robot interactions for the two tasks. In Figure 6, the robot is performing the motor commands corresponding to the sentence “Point the guitar before you put on the left the violin.” (A) the robot is pointing the “guitar” (blue object), (B) the robot is finishing the displacement of the “violin” (red object). In Figure 7, the robot has to describe the scene relative to the object of focus: (A) the user sets the object of focus in the scene, where other objects are already present; (B) the robot is describing the position of the focus object relative to the other objects.

In the following subsections we describe events and human-robot interactions during testing mode. These descriptions correspond to the transcript of sections of videos obtained during experiments with the Action Performer and Scene Describer tasks. Videos are available in the supplementary materials.

### Trials for execution of action performing task

For illustrating the Action Performer task, we will use the same initial positions for the objects across trials, and vary the oral description made by the user. The initial positions of objects are the following: the guitar is on the right and the violin is on the left. Absolute and relative positions are defined relative to the user. For the first trial, the action the user asked the iCub to perform was *put (violin, left)*. The user told the robot the non-canonical sentence 1 in Table 3. The robot then put the violin on the left. For trials 2–4, the desired first action was *point (guitar)* and the second action was *put (violin, left)*. The robot first pointed to the guitar and then put the violin on the left. In this way, we could test for different ways of saying the same meaning but with different grammatical forms of sentences (i.e., with different grammatical constructions). For each sentence spoken to the robot, we indicate in Table 3 for clarification the type of sentence (canonical or non-canonical word order) and the order of actions (chronological or non-chronological order of actions).

**Table 3:** **Example of sentences used during testing mode of action performer task**.

**#**	**Sentence**	**Type**
1	On the left put the violin	Non-canonical
2	After you point the guitar put the violin on the left	Canonical, chronological
3	Before you put the violin on the left point the guitar	Canonical, non-chronological
4	Point the guitar before you put on the left the violin	Canonical and non-canonical, chronological

### Trials for scene description task

For the Scene Describer task, the user was first asked to set the initial position of the objects (this is indicated by the robot when it says “set objects”). Then the user was asked to identify the focus object (indicated by “focus”). The latter is what should be in focus in the sentence, i.e., the subject (i.e., the agent in this case) of the sentence. After that, the robot asked the user if it should produce a usual (i.e., canonical) or non-canonical sentence. Then the robot processed all the information and described the scene by producing a sentence (“robot sentence”). See Table 4 to see a subset of trials for the Scene Describer task.

**Table 4:** **Subset of trials for the scene describer task**.

**Trial**	**Set objects**	**Focus**	**Sentence type**	**Robot sentence**
1	Guitar middle	Trumpet right	Canonical	The trumpet is to the right of the guitar
2	Trumpet middle	Guitar left	Non-canonical	To the left of the trumpet is the guitar
3	Guitar left, trumpet right	Violin middle	Canonical	The violin is to the right of the guitar and to the left of the trumpet
4	Guitar left, violin right	Trumpet middle	Non-canonical	To the left of the violin and to the right of the guitar is the trumpet

Set objects indicates the position of initial object(s) on the tactile table. Focus indicates the object that is put on the table when the “focus object” is asked by the robot. Sentence-type indicates the type of sentence that should be generated. Robot Sentence indicates the corresponding sentences produced by the robot.

In order to get an appreciation for the near real-time behavior of the system, we examined experimental data logs and collected data from 22 experiments with the scene describer and from 66 experiments with the action performer.

The execution times for the Scene Describer task are recorded from when the subject places the objects on the table, until the system responds with the description. This includes file transfer time from the Supervisor to the neural network model, and back, along with the model processing. Despite these delays, the total time of execution is around 30 s, which is near-real time performance. Likewise, for the action performer, processing of the spoken sentence by the model takes place within approximately 20 s, and then the execution of the actions by the robot is slower. This long time for executing actions is due to (a) safety limits on velocity, and the fact that (b) many of the commanded actions include two distinct actions. Still, from spoken command to completed action, the execution is less than a minute, again, within the bounds of near-real time performance.

Looking in more detail at the time used by actually running the neural network, we measured the time from sending the file to the network, to the time to retrieve the file containing the actions to be sent to the robot. For 66 trials of the AP task this required on average 6.02 s (SD ± 0.33 s), and for 22 trials of the SD task the file transfer and neural network execution required 9.42 s (SD ± 0.96 s). This can be considerably improved by replacing the file-based communication with a client-server communication in the YARP framework.

### Neural output activity of the models

In this section we will illustrate the activity of the neural network model for the two tasks. One has to recall that the output of the neural network is used to generate the behavioral and spoken responses.

#### Comprehension model neural activity for action performer task

**Figure 8:**
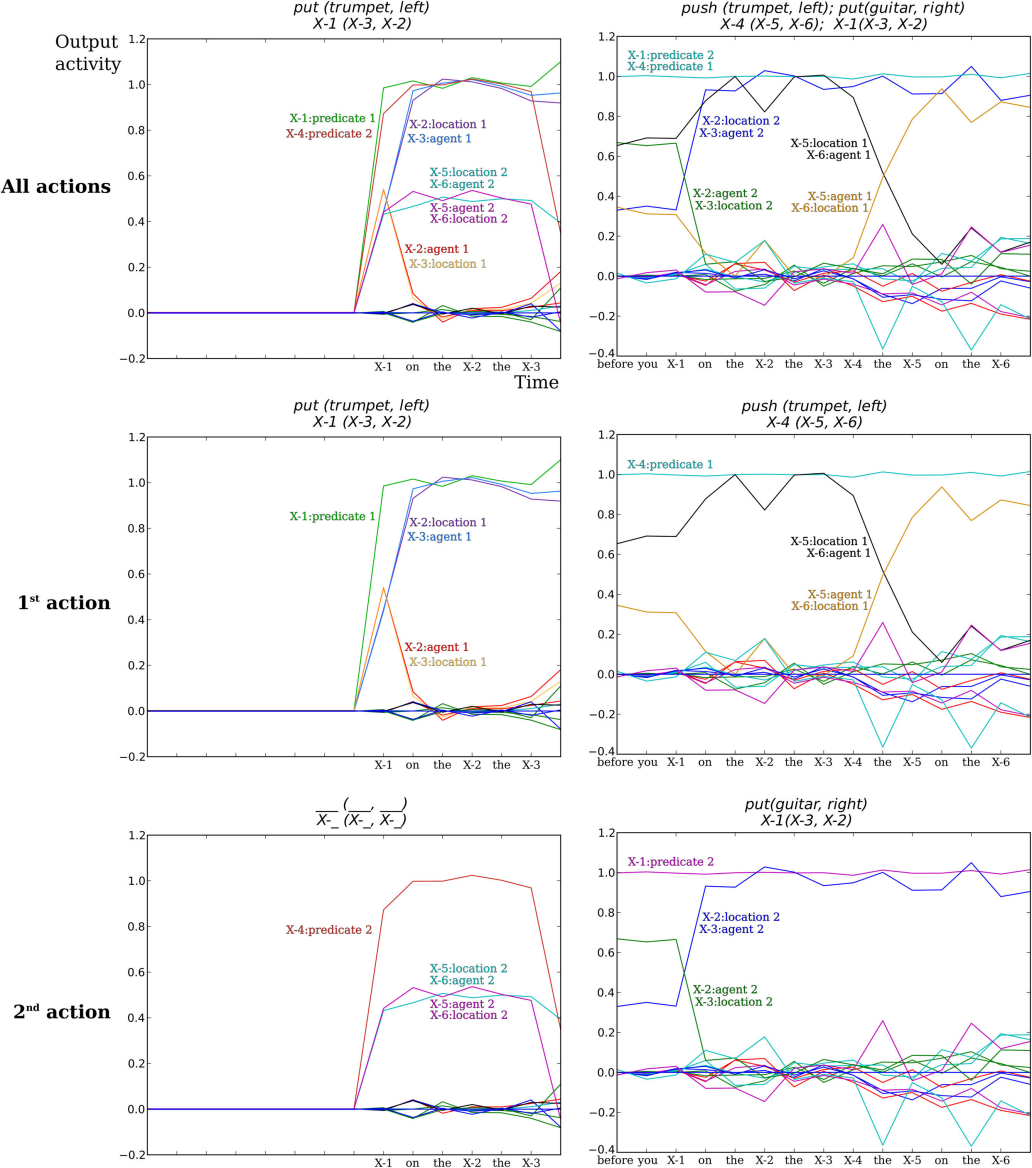
**Example of output activity of the comprehension neural model for the “Action Performing” task**. Each colored line indicates the pseudo-probability for each semantic word to have a given role (*predicate*, *agent*, *location*) for each of the two specified actions. (Top row) Output activity for both actions. (Middle row) Output activity for the first action to perform. (Bottom row) Output activity for the second action to perform. (Left column) The input sentence was “put on the left the trumpet.” The model correctly determines that there is only one meaning-predicate *put (trumpet, left)*. X-1, X-2, X-3 … indicate the 1st, 2nd, 3rd, … SW markers. For X-5 and X-6 plots are superimposed, as the output neurons “X-5:location2” and “X-6:agent2” have the same activity for this sentence. The labels of SW and meaning are thus empty in the lowest left panel because there is no second action in this sentence. (Right column) The input sentence was “before you put on the right the guitar push the trumpet on the left”: the model correctly determines the two meanings in the right order *push (trumpet, left)* and then *put (guitar, right)*. Several curves are also superimposed.

In Figure 8 we illustrate the output activity for two example trials on the Action Performer task. Recall that each word is coded as a square wave of 1 time step. From the beginning of the input of the grammatical construction, the read-out activity starts to change and is updated each time a new word is presented in input. This activity can be interpreted as an estimated prediction given the inputs thus far. These estimations are based on the statistics of the sentence forms of the training corpus (see Hinaut and Dominey, 2013, for details). In the left columns of Figure 8, the model correctly determines that there is only one meaning-predicate which is *put (trumpet, left)*. We see that at the last time step the neural activations concerning the on-going predictions on a potential 2nd predicate-meaning all fall below the threshold of 0.5, and as a consequence only one predicate-meaning is considered.

In some cases, this activity can be used to know the correct response before the end of the sentence. In future experiments, this could potentially allow the robot to start moving the object before the end of the sentence. This is actually a behavior that seems natural in human interaction when one give the other a series of orders. When the first order is given the human can start to do the 1^st^ action while listening to the rest of the orders (for instance when someone lists what has to be done for a cake recipe, while another one is making the cake).

For the Action Performer task, we show the activity for sentences that were not learned (i.e., not seen beforehand). Constructions shown in Figure 8 where not in the training data, but only in the test data. Even though the constructions were not pre-learned, the model was still able to correctly recognize them, demonstrating generalization capabilities. For more information on the model generalization performances see (Hinaut and Dominey, 2012, 2013)

#### Production model neural activity for scene description task

**Figure 9:**
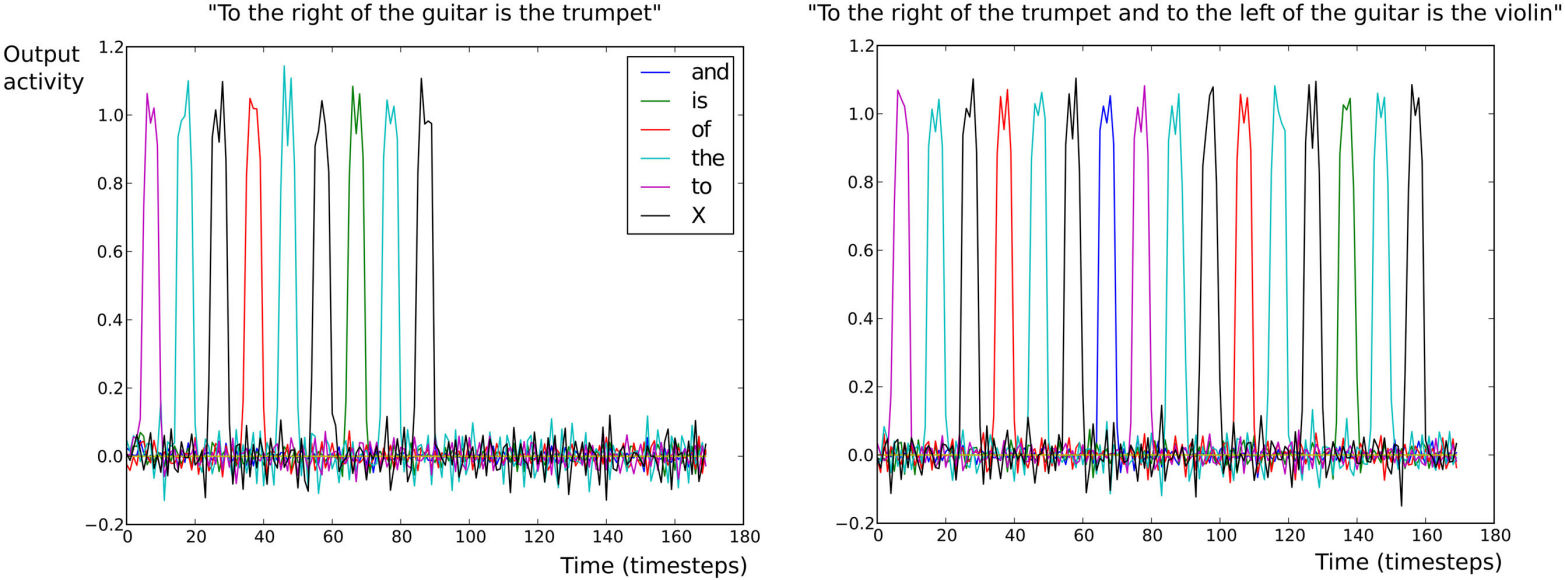
**Output (read-out) unit activations of the production neural model in the Scene Description task**. Each colored line represents a different read-out neuron. Each read-out neuron corresponds to a different word: either a grammatical word or a SW marker. On the x-axis is indicated the number of time steps. On the y-axis is indicated the neural activity for output neurons. X indicates the semantic word (SW) marker. (Left) The construction found is “To the X of the X is the X.” The sentence correctly recovered after replacement of the SW markers is “To the right of the guitar is the trumpet.” (Right) The construction found is “To the X of the X and to the X of the X is the X.” The sentence correctly recovered after replacement of the SW markers is “To the right of the trumpet and to the left of the guitar is the violin.”

Figure 9 illustrates the readout unit activations for two different meanings and different sentence forms in the Scene Description task. In the left panel of Figure 9, the meaning given in input was *right (trumpet, guitar)* with the sentence form *SW1(SW3, SW2)*. The model correctly generated the sentence “to the right of the guitar is the trumpet.” In the right panel of Figure 9, the meaning given in input was <*right (violin, trumpet)*, *left (violin, guitar)*> with the sentence form <*SW1(SW5, SW2)*, *SW3(SW5, SW4)*>. The model correctly generated the sentence “to the right of the trumpet and to the left of the guitar is the violin.”

These results indicate that the system works correctly in the SD and AP tasks, under controlled conditions. We should also evaluate the capacity of the system to accommodate less controlled conditions. In Hinaut and Dominey (2013) we addressed the generalization capabilities of the sentence comprehension model with large corpora (up to 90 K sentence-meaning pairs). In the current research we demonstrate that the model can learn and reuse grammatical constructions for sentence production. The extensive investigation of generalization properties (including the analysis of “incorrect” generated sentences) is beyond the scope of the current paper, and will be the subject of future research.

### Action performing training with naïve subjects

Here we report on the results of the Action Performer model, when trained and tested with a set of sentences from five naïve subjects. Examination revealed that several additional closed class words were used by our subjects. They were used to define the set of possible inputs to the model. Here we defined the list of closed class word in a simple and systematic way: all the words that were not in the meaning part of the <sentence, meaning> pairs (i.e., the open class words that had a thematic role to be find) were defined as closed class words. Some of these words may appear once or a few times in the corpus, thus it is difficult for the model to learn their function. Please refer to Supplementary Material SM3 for the extended list of all 86 closed class words.

#### Naïve subject corpora

From the initial corpus of 380 sentences, a new corpus, where 7 <sentences, meaning> pairs were eliminated, was created: we will call the latter the 373 corpus. These seven sentences did not fulfill the minimal conditions in order to be processed correctly by the system: they were ill-formed. For instance (1) they did not describe the actions properly (e.g., “make a U-turn”: invention of new actions instead of using the atomic actions proposed), or (2) they did not refer to the objects by their name (e.g., “touch both objects”). As we will see in the learnability analysis, these seven sentences were part of the sentences that were not learnable by the system (see learnability test). The following analyses were performed on both initial and 373 corpora (see supplementary material SM4 to see all the <sentence, meaning> pairs of both corpora).

#### Learnability test

The analysis of the naïve subject data proceeded in two steps: learnability and generalization tests. We first tested the learnability capability of the complete set of sentences for each corpus: the reservoir was trained and then tested on the same set of sentences. Because of the increase in size and complexity of the training corpus compared to experiment 1—subjects were ask to provide complex sentences structures—, we increased the reservoir size from 100 to 500 and 1000 neurons (for the generalization test). For the learnability test specifically, we deliberately took a large number of neurons (3000) in order to be sure that the system could learn the maximum <sentence, meaning> associations possible. Sentences are considered learnable if they were correctly learned “by heart” (i.e., without generalization) by the system. The learnability test results are taken as a reference for the generalization tests. The learnability test is based on the hypothesis that if the system is not able to learn some <sentence, meaning> associations by heart, then the system would not be able to generalize to such sentences. Thus, the error obtained in the learnability test should be the lowest possible.

For the learnability test we created four instances of the model (i.e., different random generator seeds were used to generate the weight connections), but there is no variability between the results of these instances—learnable sentences are the same. Results for this learnability capability are illustrated in the “Learnability” column in Table 5. Only 16 sentences of the entire initial 380 corpus (i.e., 4.21%) were considered not learnable. Thus, the vast majority of utterances produced by the naïve users were exploitable and learnable. This confirms the viability of the approach. For the 373 corpus, learnability error falls to 2.41% with only nine sentences that are not learnable. These few sentences are not learnable because there is a competition between them that “interferes” because of an existing ambiguity among them. For example, two sentences can have the same surface form but correspond to two different constructions. If one of these constructions is more frequent than the other in the corpus, the other will not be learned, because the model will always select the most probable mapping for a given structure. An example of such ambiguity is seen in our corpus due to the use of “irrelevant” information in the sentence: “point the circle on my left” has the meaning *point(circle)* so “left” is irrelevant in this sentence, but the sentence “put the cross on my left” has the meaning *put(cross, left)* so left is important in this one. Because the first type of structure (see sentences 152, 190, and 192 in SM4.1) has been used more frequently by the users than the second type (155, 156), the most frequent “desirable” behavior of the system is to ignore the open class word coming after “on my.”

**Table 5:** **Learnability and best generalization capabilities on the naïve subject initial 380 corpus**.

	**Learnability error**		**Generalization error (best)**	
**Global**	16/380 (4.2%)		133/380 (35.0%)	
	**Single action**	**Double action**	**Single action**	**Double action**
**Simple sentence**	4/25 (16.0%)	9/165 (5.5%)	2/25 (8.0%)	44/165 (26.7%)
**Elaborate sentence**	0/25 (0.0%)	3/165 (1.8%)	9/25 (36.0%)	78/165 (47.3%)

(Left) Learnability test performed with a reservoir of 3000 neurons. Number of non-learnable sentences for different sentence categories. For each category, the number of non-learnable sentences is divided by the total number of sentences for that category, with the corresponding percentage in parentheses. Only 4.2% of sentences are not learnable: this indicates that most of the corpus is learnable. (Right) Best generalization errors for different sentence categories. For each category the neural model is able to generalize to some not learned sentences. As one could expect, generalization performances are better for Simple sentences than for Elaborate sentences. These results were obtained for a model of 1000 neurons using LoO method. No variability is observed when using such a number of neurons: the sentences that fail in generalization are always the same.

This learnability test is important to demonstrate the difficulty of the task, and it constitutes a preliminary step before looking at the generalization capability; because sentences that are not learnable have a priori no chance for being part of the group of sentences that the neural system could “understand” (i.e., generalize on). Of course the learnability of a sentence is also dependent on other sentences in the corpus: in this view, if one sentence is not learnable, it means that it is an outlier in this corpus.

#### Generalization test

In a second step we tested the ability of the model to generalize to sentences not used in training. We used a standard “leaving one out” (LoO) method: the model is trained on nearly all sentences and then tested on the sentence left out of the training data. This corresponds to the case were the robot-neural system has been taught hundreds of sentences and we want to test its ability to understand correctly a new sentence given by a naïve user. Even if that new sentence has a grammatical structure different from those in the training set, the system could nevertheless generalize to this untrained structure in some cases; this was demonstrated in Hinaut and Dominey (2013). For this study, we used two sizes of reservoirs: 500 and 1000 neurons. We run 10 instances of the model for each size and each corpus.

For a reservoir size equal to 1000 units using the initial 380 corpus, 133 sentences failed to pass the generalization (LoO) test in all 10 simulations (i.e., for all 10 instances). We can consider that, for this amount of units in the reservoir (1000)—related to the computational power of the system—the corpus did not enable the system to have sufficient grammatical information to allow generalization to these 133 sentences. In Table 5, best generalization errors for different sentence categories are provided in the left column. The best generalization error over all categories is 35.0%. As expected, generalization error increase from Single to Double action sentences, and from Simple to Elaborate sentences. These results were obtained for a model of 1000 neurons also using LoO method. Considering the learnability results, which could lead to only 8.0% error for Simple—Single Action category, the system displays a good ability to generalize to unseen sentences. In particular, for the simple sentences (both single and double actions) the system is able to generalize to more than 75% of unseen sentences: this is an important result as in a natural conditions subjects will tend to produced spontaneously this type of sentences (that we categorized as “Simple”).

#### Discussion on the “utility” of the learnability test

In Table 5, one could remark that for the Simple Sentence—Single Action category a lower error is obtained for the best generalization than for the learnability. This could be explained by the fact that LoO results with 1000 neurons are the “best” accumulated over 10 simulations, thus it is possible that sometimes a given sentence that could not be learnt by heart with a reservoir of 3000 units (when nearly the whole corpus is correctly learned), could be generalized when using a smaller reservoir—here 1000 units—(when only a part of the corpus is correctly learnt). In particular if there is a “competition” between some sentences in the corpus that lead to an ambiguity. Consider that a group of sentences with a given construction A could not be learnt simultaneously with sentences with construction B; if more sentences of group A than sentences of group B are learnt correctly, then the system could not learn (or generalize) correctly to sentences of the concurrent group B, and vice versa.

Indeed, this partly contradicts the hypothesis that was at the origin of the learnability test, because of sentences that could not be learnt with this test. However, the “best” generalization results are not obtained with a single reservoir, but with 10 reservoirs running in parallel using the best possible combination of results of each reservoir—such an optimal combination may not be found without knowing in advance which reservoirs give the best answer for each sentence. Consequently, this is a demonstration that the learnability and generalization of a sentence is dependent on the corpus it constitutes, as the learning system tends to learn the corpus coherently. Thus, outlier constructions that have poor chance to be learnt, which is a useful property if possibly ungrammatical constructions are present in the corpus. Here a part of ungrammaticality could also be interpreted as “less frequent,” because for a learning system what makes a construction learnable (i.e., grammatical) is the fact that it has a higher probability of occurrence.

#### Summary of results for the generalization test

In Table 6 can be seen a summary of generalization errors for different conditions. A bigger reservoir (1000 compared to 500 neurons) clearly demonstrates better performances. On the contrary, the corpus does not have much influence on the performances. One can see that when the number of neuron increases, the negative influence of ill-formed sentences, removed in the 373 corpus, tend to decrease. Looking at the best error values, obtained when counting only errors that are made in common by all 10 instances, there is a clear decrease compared to the mean values. This big difference between best and mean values shows that there is a high variability regarding which sentences are recognized by the different reservoir instances. This indicates that there is a clear potential to increase the performance of the system by combining several reservoirs in parallel. In addition, even better performance could be found by increasing the number of units in the reservoir, but this is not the point of the current study. We did not explore for the best parameters of the reservoir, we considered that the parameters we found in Hinaut and Dominey (2013) were sufficiently robust to be applied to a new type of corpus—produced by naïve users, demonstrating in this manner the robustness of this reservoir language model.

**Table 6:** **Generalization errors**.

		**Initial corpus**	**373 corpus**
**500 N**	**Mean (std.)**	70.13 (1.87)	68.96 (2.03)
	**Best**	46.05	44.50
**1000 N**	**Mean (std.)**	58.53 (2.23)	58.26 (1.37)
	**Best**	35.00	34.85

Results for different conditions are shown: initial and 373 corpus and reservoir sizes of 500 and 1000 neurons. For each condition, the average error (Mean) over 10 instances along with the standard deviation (std.), and the best error (counts of only the errors in common within the 10 instances) are indicated. One can see that when the number of neuron increases, the negative influence of ill-formed sentences, removed in the 373 corpus, tend to decrease.

#### Some remarkable properties of the flexibility of the system

Some of the sentences that produced successful generalization are worth noting. Sentences (230), (245), (260), and (268) (see Table 7) illustrate the use of the impersonal pronoun “it” in various configurations of distinct constructions. Processed as a closed-class (i.e., grammatical) word, “it” indicates the appropriate role for the referent open class (i.e., semantic) element: the system is able to generalize correctly the function of the grammatical word “it” and bind to the correct role the semantic word it refers to. In a sentence like (230) (see Table 7) the second semantic word “circle” will be considered as the “object” of both actions, “grasp” and “point.” Sentence (92) illustrates a similar situation, where the closed class word “twice” informs the system that the same action is repeated two times. Thus, in a certain sense, the system has learned the non-trivial meaning of the word “twice.” Similarly, in sentence (313) this special function can be learned even when relying on several words: “two times.” The system also acquires non-trivial use of the temporal relatives “before” and “after.” In (198), (214), and (340), “before” is used in such a way that the first action appearing in the sentence is actually to be performed second. Thus, in these situations, the presence of “before” results in a temporal inversion of the commanded actions. Interestingly, the system can also master a different use of “before” as illustrated in (5): here “before” does not result in an inversion, the order of actions in the sentence is preserved in the execution of actions. Similarly in sentence (268), “after” plays also the role of temporal inversion. Moreover such sentence illustrate how these different properties—“it”: reference, “after”: inversion—can be combined. Sentence (340) has a particular structure: “before” is the unique closed class word present in the sentence, the four open class words follow in a row. The system is nevertheless able to learn correctly this structure even if it could not distinguish the different open class words from one to another, because it does not have access to the semantics of these words. Sentences (198) and (214) have also the particularity to have useless closed class words—for the given task—“please” and “you” have no specific function, but the model still has to learn to ignore these words. Although the system has not been designed to reach this level of “interpretation” of closed class words, it is able to generalize its use in not learned sentences. This ability of the system to work with non-predefined cases demonstrates its flexibility.

**Table 7:** **Example sentences produced by naïve subjects (of the 373 corpus; see SM4.2), and understood by the model (i.e., 0% error in LoO generalization simulations for a reservoir of 1000 units)**.

(5) Point the triangle **before** grasping the circle
(20) Put the cross to the left **before** grasping the circle
(92) Point to the cross **twice**
(198) **Before you** grasp the cross **please** grasp the triangle
(214) **Before** pushing the triangle to the middle **please** push the cross to the right
(230) Grasp the circle and then point to **it**
(245) Touch the triangle then move **it** to the left
(260) The cross touch **it**
(268) Point to the circle **after** having grasped **it**
(313) Point cross **two times**
(340) **Before** grasp circle point triangle

Closed class words indicated in bold have a specific function and the system has to learn it without any additional feature to treat these special words. These words are common words of natural language, but they are not essential to form a correct sentence understandable by the system. Nevertheless, the system is able to learn their specific function. Numbers in parenthesis indicate the identifiers of the sentences for the corpus 373; note that identifiers are not the same for initial and 373 corpora.

## Discussion

Neural inspired models of sentence comprehension and production have been extensively developed (e.g., McClelland et al., 1989; Elman, 1990; Miikkulainen, 1996; Christiansen and Chater, 1999; Chang, 2002; Dominey, 2002; Dominey et al., 2003; Tong et al., 2007; Takac et al., 2012). What is novel here is that the model we use is a neural network model of language comprehension and production that has very little language-related knowledge pre-coded (only the semantic/grammatical word distinction which has been demonstrated to be learnable), and that can learn and integrate new constructions during on-line interactions. This on-line learning contributes to a new level of flexibility in human-robot interaction, as new constructions can be added to the inventory during an ongoing interaction.

Previous research has used language to command humanoids (e.g., Dominey et al., 2007; Lallée et al., 2012; Petit et al., 2013), and to allow robotic systems to describe actions (Dominey and Boucher, 2005b). The current research for the first time demonstrates real-time acquisition of new grammatical constructions for comprehension and production that can be used respectively in commanding the robot and in asking the robot to describe the physical world. This is of interest both in theory and in practice. In theory, it demonstrates that the form-to-meaning mapping that we have employed in learning grammatical constructions can be used in both directions, to generate meaning from form, and to generate form from meaning. In practice, this means that the system can adapt to individual differences in the way users employ language to describe and request actions. The current research also addresses how language can allow for the coordination of multiple sub-actions in time, using the prepositions “before,” and “after.” Learning of these terms has a long history of research in child language development, and it poses an interesting problem because of the interaction with non-temporal event ordering and non-canonical syntactic structure (Carni and French, 1984). Our work can contribute to the debate by indicating that a system that is sufficiently powerful to handle canonical and non-canonical events in single and double event sentences can do the same in sentences in which order is expressed with prepositions including “before” and “after.” Interestingly, the key assumption is that these prepositions are processed in the model as closed class or grammatical words, which can then directly contribute to the elaboration of the form to meaning mapping.

Because of this flexibility, the framework that we have developed potentially enables naive users to interact with the robot, indeed there is no “predefined” way of giving a command or description of an action such as *put (toy, left)*; the user could say “put the toy on the left” or “on the left put the toy.” In this way, we are able to escape from a 1-to-1 sentence-action correspondence: several sentences could indicate the same meaning.

Concerning the production model we partly escape the 1-to-1 sentence-action (or sentence-scene) limitation because we can specify if we want a canonical or non-canonical sentence type. We could specify a more precise sentence type, for instance by specifying the semantic word of focus. But this problem could be tackled in a more general way. In order to be able to generate several sentences with the same meaning, we could consider 2 alternatives. (1) We could add feedback connections from the readout layer to the reservoir with the addition of noise either in the reservoir states or in these feedback connections. Thus, the network would not produce every time the same pattern of words, but different ones. The noise would enable the network to be driven by one of the possible learned sentences (word patterns). (2) Use an additional self-organization map (SOM) based on the semantic words. During training this SOM will tend to organize words that appear in the same sentences in the same area of the map. During testing, the SOM activation will provide a supplementary input to the sentence production model in order to give a kind of context and enable the model to generate one pattern of words that is context relevant. In this way, if some sentence constructions are commonly used with certain semantic words, it will produce the more common sentences. Both alternative solutions may enable the production of constructions that were not learned, i.e., give the production generalization capabilities (like the comprehension model). Finally, the generation of different non-canonical forms allows the system to manipulate the grammatical focus while describing the same situation, as illustrated in Table 1.

The production model introduced here is able to learn to produce grammatical constructions when given the meaning, coded in the same way that the comprehension model output is coded. This is the first time that we demonstrate that the input and output of the comprehension model could be reversed in order to do the “inverse” task (i.e., production instead of comprehension). This is an interesting property that may be useful in further understanding human language. Indeed, we have here a system that is able to do grammatical construction comprehension and production with a common coded meaning representation (which corresponds to the output of the comprehension model, and to the input of the production model). We can imagine that the two models can be running in parallel, with the outputs of the production model connected to the inputs of the comprehension model. In this way, when the production model would be generating a sentence, the latter could be decoded in real-time and fed to the inputs of the comprehension model. Thus, the comprehension model will reconstruct in real-time the meaning of the sentence produced by the production model. Consequently this would allow the system to check if the produced sentence is correct or not to the original meaning (i.e., the input of the production model). A correction mechanism could be then added to compensate when errors of productions are made. Such a correction mechanism appears to exist in human language behavior, as when one notices that they have produced a word instead of another in the middle of a sentence, they correct their sentence production in real-time accordingly. Detection of such a production error would likely be accompanied by specific brain response, as it is the case for the P600 event related scalp potential when an ungrammatical word or complex sentence is processed. In a previous study using our comprehension model (Hinaut and Dominey, 2013) we showed that a kind of instantaneous derivative of the output values—the sum of absolute change of all outputs—could be related with a P600-like event. In the reverse sense, the output of the comprehension model could be input to the production model, allowing the listener to predict the upcoming words of the speaker. Another alternative would be to combine both comprehension on production within a same model, with feedback connections from both discovered thematic roles and produced words: a unique reservoir would do both tasks at the same time; this would probably require an online learning algorithm.

We collected data from 66 successful runs of Experiment 1 (action performer) and 22 successful runs of Experiment 2 (scene describer), with experienced users, in order to estimate the timing of the interactions. We did not focus on error analyses, but these data already indicate that the system functions reliably. The next step was to see how this would translate sentences with variability inherent to “naïve” users. This gave the results we presented in Experiment 3. Experiments 1 and 2 demonstrated that the comprehension and production models could function in the HRI setting. Given this validation in the HRI setting, Experiment 3 then tested the model with input from naive subjects, in the context of interaction in the shared environment of the ReacTable. This experiment with the naïve subjects is particularly interesting, as it provides the model with a form of “cognitive variability” in the language used, which goes beyond that employed when “insider” researchers interact with the robot. The use of the impersonal pronoun “it,” words like “twice,” the use of “before,” and “after,” in the diverse configurations allowed a test and finally an illustration of the adaptability of the language model. The good learnability of the sentences—93% of the corpus is learnable—indicates that the naïve subjects can make really complicated sentences that may contain only partial information. The relatively robust generalization, particularly for the “simple” sentences (>75% generalization) indicates that the model was able to extract the relevant information from this relatively small (<400 sentences) corpus; it also indicates that the naïve subjects are “playing the game,” i.e., they are attempting to speak in a reasonable way to the robot in the “simple” sentence condition. Future research should asses directly how the robot will interact with naïve subjects over extended time.

### Conflict of interest statement

The authors declare that the research was conducted in the absence of any commercial or financial relationships that could be construed as a potential conflict of interest.

## Supplementary material

Video demonstration of the scene description in Experiment 1 can be seen at: http://youtu.be/AUbJAupkU4M

Video demonstration of the action performer in Experiment 2 can be seen at: http://youtu.be/3ZePCuvygi0

SM1. Input file for Action Performer task with train and test data used for Figure 8.

SM2. Input file for Scene Description task with train and test data used for Figure 9.

SM3. Details concerning the Naïve Subject Experiment: List of closed class words used and removed sentences from raw corpus for the naïve subject experiment; List of the 7 <meaning, sentence> pairs removed from the raw corpus of 380 sentences (i.e., sentences that are not present in 373 corpus).

SM4. Files containing the detailed results of the naïve subject experiment for the learnability test for both corpora (initial or 373 corpus) with a reservoir size of 3000 units. (See data sheet files 4–6.)

SM5. Files containing the detailed results of the naïve subject experiment for the generalization test for the different conditions: initial or 373 corpus, and 500 or 1000 reservoir units. (See data sheet files 7–10.)

SM6. Movie scenes shown to the users for the naïve subject experiment.
