# Supplementary material for: Exploring the acquisition and production of grammatical constructions through human-robot interaction with echo state networks
Source: Front Neurorobot. 2014 May 6;8:16. doi: 10.3389/fnbot.2014.00016 (PMC4018555; doi:10.3389/fnbot.2014.00016)
Supplement: Supplementary file 1 [file DataSheet1.DOC]

<train data>

#############################################

### Data used for training.

### Each line is coded in the following format: "meaning ; sentence # commentaries"

### Lines or end of lines after character # indicate commentaries that are not taken into account by the file parser.

#############################################

#############################################

### 1 action ###

### Word order is expressed relative to the order of Predicate (V - verb), Agent (S - subject) and Location (O - Object).

put trumpet left;put the trumpet on the left # 1R/C (canonical - VSO)

put trumpet left;on the left put the trumpet # 1R/N'1 (OVS)

## This line in not used for training, but used for testing ## put trumpet left;put on the left the trumpet # 1R/N'2 (VOS)

put trumpet left;the trumpet put it on the left # 1R/N'3 (SVO)

#~~ (SOV) too ambiguous

#~~ (OSV) too ambiguous

## This line in not used for training ## grasp guitar;grasp the guitar # 1R/C (VO)

grasp guitar;the guitar grasp it # 1R/N (OV)

#############################################

#############################################

### 2 actions ###

### A canonical (VSO), B canonical (VSO)

# A:push the trumpet on the left , B:put the guitar on the right

push trumpet left,put guitar right;push the trumpet on the left and then put the guitar on the right # A(and then)B #

push trumpet left,put guitar right;after you push the trumpet on the left put the guitar on the right # (after you)A,B

push trumpet left,put guitar right;push the trumpet on the left before you put the guitar on the right # A(before you)B

push trumpet left,put guitar right;before you put the guitar on the right push the trumpet on the left# (before you)B,A

### A non canonical'2 (VOS), B canonical (VSO)

# A: push on the left the trumpet , B: put the guitar on the right

push trumpet left,put guitar right;push on the left the trumpet and then put the guitar on the right # A(and then)B

push trumpet left,put guitar right;after you push on the left the trumpet put the guitar on the right # (after you)A,B

push trumpet left,put guitar right;push on the left the trumpet before you put the guitar on the right # A(before you)B

push trumpet left,put guitar right;before you put the guitar on the right push on the left the trumpet# (before you)B,A

### A canonical (VSO), B non canonical'2 (VOS)

# A: push the trumpet on the left, B : put on the right the guitar

push trumpet left,put guitar right;push the trumpet on the left and then put on the right the guitar # A(and then)B

push trumpet left,put guitar right;after you push the trumpet on the left put on the right the guitar # (after you)A,B

push trumpet left,put guitar right;push the trumpet on the left before you put on the right the guitar # A(before you)B

## This line in not used for training, but used for testing ## push trumpet left,put guitar right;before you put on the right the guitar push the trumpet on the left# (before you)B,A

### A non canonical'2 (VOS), B non canonical'2 (VOS)

#A: push on the left the trumpet , B : put on the right the guitar

push trumpet left,put guitar right;push on the left the trumpet and then put on the right the guitar # A(and then)B

push trumpet left,put guitar right;after you push on the left the trumpet put on the right the guitar # (after you)A,B

push trumpet left,put guitar right;push on the left the trumpet before you put on the right the guitar # A(before you)B

push trumpet left,put guitar right;before you put on the right the guitar push on the left the trumpet # (before you)B,A

#############################################

</train data>

<test data>

#############################################

### Data used for training.

### Each line is composed only of a sentence, the meaning has to be retrieved by the neural network.

#############################################

put on the left the trumpet

before you put on the right the guitar push the trumpet on the left

</test data>
