# Supplementary material for: Exploring the acquisition and production of grammatical constructions through human-robot interaction with echo state networks
Source: Front Neurorobot. 2014 May 6;8:16. doi: 10.3389/fnbot.2014.00016 (PMC4018555; doi:10.3389/fnbot.2014.00016)
Supplement: Supplementary file 2 [file DataSheet2.DOC]

<train data>

#############################################

### Data used for training.

### Each line is coded in the following format: "meaning ; sentence # commentaries"

### Lines or end of lines after character # indicate commentaries that are not taken into account by the file parser.

#############################################

#############################################

### 1 spatial relation ###

#left violin trumpet;the violin is left of the trumpet

left violin trumpet;the violin is to the left of the trumpet # Canonical

left violin trumpet;to the left of the trumpet is the violin # Non canonical

#############################################

#############################################

### 2 spatial relations ###

left violin trumpet,right violin guitar;the violin is to the left of the trumpet and to the right of the guitar # Non canonical

left violin trumpet,right violin guitar;to the left of the trumpet and to the right of the guitar is the violin # Non canonical

#############################################

</train data>

<test data>

#############################################

### Data used for testing.

### Each line is coded in the following format: "meaning : sentence-type"

### sentence-type could be canonical (usual) or non-canonical (non-usual).

### The grammatical structure (i.e. sentence) with the corresponding sentence-type has to be generated by the neural network.

### N indicates that we want a non-canonical sentence (C would indicate a canonical sentence)

### This non-canonical sentence has a predefined semantic word order.

#############################################

right trumpet guitar : N

right violin trumpet,left violin guitar : N

</test data>
