# Supplementary material for: Exploring the acquisition and production of grammatical constructions through human-robot interaction with echo state networks
Source: Front Neurorobot. 2014 May 6;8:16. doi: 10.3389/fnbot.2014.00016 (PMC4018555; doi:10.3389/fnbot.2014.00016)
Supplement: Supplementary file 3 [file DataSheet3.DOC]

*** List of 86 close class words used for Naïve Subject Experiment:

a, after, afterwards, again, all, also, and, around, as, at, back, before, blue, both, but, by, can, could, directions, do, done, empty, finally, finger, fingers, finished, first, firstly, followed, going, hand, have, having, in, index, is, it, itself, my, need, next, not, object, objects, on, onto, once, over, palm, place, please, red, row, same, second, should, slowly, space, start, steps, table, that, the, then, there, thing, things, this, time, times, to, too, twice, two, way, well, weve, when, with, u-turn, unique, using, you, your, youre, youve

*** List of <meaning, sentence> removed from the raw corpus of 380 sentences of Naïve Subject Experiment (i.e. sentences that are not present in 373 corpus):

(format: sentence identifier; correct meaning; sentence)

180; push middle , put right; push the blue object to the middle and then put it to the right again

184; put cross right , push cross right; put the cross to the right and do a u-turn

187; put circle right , put cross right; put both objects to the right

188; put triangle middle , put triangle left; put the triangle to the right in two steps

200; hit circle , point circle; hit and point the unique object on the table

218; push circle middle , put circle right; push the blue circle to the right and then go back

224; push triangle left , push cross left; push both objects to the left
